# Expected Results from a Predictable Phenomenon: An Approach to Education in Times of Pandemics

**DOI:** 10.1017/dmp.2020.442

**Published:** 2020-11-18

**Authors:** Nicolás Alejandro Gemelli, Luis Alejandro Boccalatte

**Affiliations:** 1 Adult Intensive Care Unit, Hospital Italiano de Buenos Aires, Buenos Aires, Argentina; 2 Head and Neck Surgery Section, Department of General Surgery, Hospital Italiano de Buenos Aires, Buenos Aires, Argentina

**Keywords:** COVID-19, education, pandemics, public health, public health surveillance

Throughout history, humans and infectious diseases have spread across the world in almost equal proportions. Since the Antonine plague, multiple epidemics and pandemics marked the course of events, pushing the capacity of the economic, political, education, and social systems to the limit, on a global scale. The paradoxical effect of outbreaks relies on their capacity to easily overwhelm the health system, destabilize the political structure, and affect the usual social behavior, but at the same time, determine great advances for mankind. The way in which we stand against these challenges determines our resiliency and preparedness to face new outbreaks. Of all the areas suffering from the effect of massive infections, education might probably be the most silent but with an enormous impact on society. An Argentinian, outdated education system, unable to adapt to constant changes, will directly affect the whole population, its activities, and its future.

In the last century, the Spanish influenza claimed millions of lives in addition to the victims of the Asian influenza, severe acute respiratory syndrome-associated coronavirus (SARS-CoV) in 2002, the influenza H1N1 pandemic in 2009, Middle East respiratory syndrome-related coronavirus (MERS-CoV) in 2012, Ebola in 2014, and, finally, the current severe acute respiratory syndrome coronavirus 2 (SARS-CoV-2) pandemic, which is registering at the moment, a total of 9 937 618 confirmed cases, 497 427 deaths, and 5 008 895 recovered patients in 188 countries. The way in which these outbreaks affect countries depends on multiple factors, and the impact is difficult to foresee. The actual numbers show that we remain unprepared, despite our attempts at planning.

Previous situations, similar to the actual health problem, have determined great advances in the medical field: the creation and extension of mechanical ventilation and the importance of vaccination after thousands of cases of poliomyelitis, the importance of safe sexual practices after HIV discovery in the 1980s, and the importance of drinkable water sources and proper sanitization of food after cholera cases in the 1990s. The 2009 H1N1 influenza pandemic, with patients suffering from severe respiratory failure and refractory hypoxemia, helped drive the expansion of extracorporeal membrane oxygenation from an infrequently used salvage therapy to a major critical care intervention.

An already globalized world together with an increase in the world’s population has encouraged infectious diseases to spread at an accelerated pace. Considering this scenario, it is not difficult to predict future outbreaks and pandemics. The predictable effect of the actual pandemic by coronavirus disease (COVID-19) took us to unexpectedly adapt to a socially distanced society. Measures that could have been taken long ago had to be suddenly implemented, not always in a completely prepared system. The current public health disaster revealed the educational unpreparedness to adapt and acquire the resources, strategies, and measures needed to stand forward. Running against time, educators, universities, and institutions had to adjust their academic programs to a completely virtual mode, improvising online classes, webinars, and exams.

Why was the impact on medical education predictable? There are several arguments to support this assertion. The first one is the feasibility of the emergence of new outbreaks in times of globalization. Ten years have passed since the last pandemic hit the world, and very little has been done to improve medical education in Argentina, proving not to be a political priority. According to preliminary data from an ongoing national questionnaire that aims to measure the impact of the COVID-19 pandemic on medical students, 3.2% of Argentine universities do not have virtual teaching activities, and 40.8% provide online lessons in just some subjects of the career. Unfavorable social factors in underdeveloped countries, heightened by the COVID-19 pandemic, show that 10.9% of medical students do not have computers and 26.6% both study and work at the same time, determining an even more complex context. While many universities continued to carry out classes through virtual platforms, there is no control over the quality of the content, teaching methods, student accompaniment strategies, future programs to fill the absence of real case-based practical work with patients.

It is important to recognize the close relationship that should exist between a country’s medical education system and the health system. On the one hand, professors of most subjects of the medical career are health professionals who carry out their activities in the context of the current pandemic, so they can be subjected to excessive working schedules and stressful situations affecting their dedication and quality toward learning spaces. On the other hand, students of the last years are normally intended to carry out their activities within the hospital units that are closed. Training areas for students, residents, fellows, and visitors are affected due to the suspension of activities in hospitals as an epidemiological measure to mitigate contagion.

It is not difficult to foresee future pandemics. We might even suspect that they will develop with even shorter time intervals between 1 and the other. During the last years, the educational trends took us to discuss many aspects of this topic, but very few things have been made in developing countries to foster and apply new techniques, to adapt to new scenarios and make a change in the educational paradigms. Efforts to use technology in favor of education could have been done long ago.

As part of a country preparedness to disaster and adverse situations, educational containment plans should be designed as a way to minimize their impact on continuing medical education.

In the last decade, information and communications technologies have been globally implemented, providing solutions in different areas. Learning spaces use a wide range of technological resources for exchanging ideas, presenting clinical cases, and complementing traditional ways of learning with interactive content. Nowadays, it is hard to interestingly approach lessons without some kind of audiovisual or virtual support. Some alternatives are suggested in Table 1.

**Table 1.** Proposed plans, resources, barriers for implementing, and possible impact in the educational field

Approach	Examples	Necessary Resources	Barriers	Impact on Education
ONLINE-BASED				
Virtual strategies, information, and communications technologies	Online campus, courses, platforms, interactive classes, case discussion sessions, breakout rooms, virtual grand rounds, telemedicine, computer-based exams, social networks, apps	Specialists in education teaching, high-speed internet access, electronic devices, IT specialists, basic computer skills, quiet learning spaces, technical support	Economic factors, limited internet access in remote areas, professionals overburdened by COVID-19 pandemic, training on the use of *telehealth*	An effective measure for guaranteeing access to education, strengthen theoretical concepts, adapting to virtual education
Assisting telehealth consults	Video call, phone call, text messages, mailing, access to medical records	Internet access, electronic devices, ethical commitment	Patient’s confidentiality, limited internet access	Learning in real situation
ATTENDANCE-BASED				
Simulation	Simulation labs, training in the use of protective equipment, role play, hands-on, virtual reality	Materials and equipment, quiet classrooms, trained staff, technical support	Expensive resources, need for adequate structure, professors’ availability	Develop practical skills, immersive learning, decision making, debriefing
Supervised patient assistance	Teaching residents and fellows, temporary licenses for advanced undergraduate students	Availability of professionals, organizing schedules	Difficult to carry out during pandemics, time-consuming, against social distancing measures	Learning in supervised real situation

It is mandatory to review education policies at levels of command to foster the creation of new and better opportunities for our students. In the best of the cases, the legacy this pandemic will leave us is the awareness of the actual state of the education system in developing countries as the essential foundations of where to start building a renewed educational structure.

